# Supplementary material for: Reward expectation drives dogs’ choices in a prosocial test
Source: Anim Cogn. 2026 May 18;29(1):50. doi: 10.1007/s10071-026-02070-4 (PMC13364881; doi:10.1007/s10071-026-02070-4)
Supplement: Supplementary file 1 — Supplementary Material 1 [file 10071_2026_2070_MOESM1_ESM.docx]

# Reward expectation drives dogs’ prosocial behaviour towards humans

**Supplemental information**

Piotti, P.^1,2^*, Spooner, R.M.^2^, Peirce K.^2^, Shelley, I.^2^, Micheletta, J.^2^, Kaminski, J.^2^

Affiliations:

^1^ Independent researcher.

^2^ Department of Psychology, University of Portsmouth, Portsmouth, UK

*Corresponding author: juliane.kaminski@port.ac.uk

# Demographic information

Table S1. Dogs’ Demographic information

| **Donor** | **Receiver** | **Sex** | **Age (years)** | **Breed** | **Donor's side** | **No. of pre-testing sessions** |
| --- | --- | --- | --- | --- | --- | --- |
| Jasper | Owner | Male | 5 | Cross | L | 4 |
| Lucca | Owner | Male | 2 | Labrador | R | 4 |
| Gus | Owner | Male | 8 | Labrador | R | 4 |
| Sailor | Owner | Male | 2 | Irish Water Spaniel | L | 6 |
| Sammy | Owner | Female | 10 | Collie | L | 3 |
| Buddie | Owner | Male | 5 | Cross | L | 8 |
| Bella T | Owner | Female | 2 | Cross | R | 6 |
| Benji | Owner | Male | 1 | Cross | R | 3 |
| Jimmy | Stranger | Male | 2 | Staffordshire Bull Terrier | L | 6 |
| Merlin | Stranger | Male | 10 | Springer spaniel | R | 6 |
| Ollie | Stranger | Male | 5 | Cross | R | n/a |
| Honour | Stranger | Female | 1 | Staffordshire Bull Terrier | L | 3 |
| Bella D | Stranger | Female | 6 | Cross | L | 2 |
| Bella F | Stranger | Female | 3 | Labrador | L | 8 |
| Groot | Stranger | Male | 1 | Labrador | R | 7 |
| Bronnie | Stranger | Male | 4 | Cross | R | 2 |

# Condition order

Table S2. Conditions’ order

| **Donor** | **Receiver** | **Test days** | **Block 1** | **Block 2** | **Block 3** | **Block 4** | **Block 5** | **Block 6** |
| --- | --- | --- | --- | --- | --- | --- | --- | --- |
| Jasper | Owner | 3 | Selfish | No-food | Pro-social | Altruistic | Pro-social control | Altruistic control |
| Lucca | Owner | 2 | Altruistic | Pro-social | Altruistic control | Selfish | No-food | Pro-social control |
| Gus | Owner | 3 | Pro-social | No-food | Selfish | Altruistic control | Pro-social control | Altruistic |
| Sailor | Owner | 3 | No-food | Selfish | Pro-social | Altruistic | Pro-social control | Altruistic control |
| Sammy | Owner | 3 | Altruistic | Pro-social | Altruistic control | Selfish | No-food | Pro-social control |
| Buddie | Owner | 3 | Pro-social | No-food | Pro-social control | Altruistic control | Selfish | Altruistic |
| Bella T | Owner | 6 | No-food | Selfish | Pro-social | Altruistic control | Pro-social control | Altruistic |
| Benji | Owner | 3 | Selfish | No-food | Pro-social | Altruistic | Pro-social control | Altruistic control |
| Jimmy | Stranger | 3 | Selfish | No-food | Pro-social | Altruistic | Pro-social control | Altruistic control |
| Merlin | Stranger | 3 | Altruistic | Pro-social | Altruistic control | Selfish | No-food | Pro-social control |
| Ollie | Stranger | 3 | Pro-social | No-food | Selfish | Altruistic control | Pro-social control | Altruistic |
| Honour | Stranger | 4 | No-food | Selfish | Pro-social | Altruistic | Pro-social control | Altruistic control |
| Bella D | Stranger | 3 | Altruistic | Pro-social | Altruistic control | Selfish | No-food | Pro-social control |
| Bella F | Stranger | 3 | Pro-social | No-food | Pro-social control | Altruistic control | Selfish | Altruistic |
| Groot | Stranger | 3 | No-food | Selfish | Pro-social | Altruistic control | Pro-social control | Altruistic |
| Bronnie | Stranger | 3 | Selfish | No-food | Pro-social | Altruistic | Pro-social control | Altruistic control |

# Descriptive statistics

Table S3. Frequency of pulling in the owner group, averaged across trials.

| **Condition** | **Median** | **min** | **max** |
| --- | --- | --- | --- |
| Selfish | 6 | 0 | 9 |
| Pro-social | 5 | 0 | 10 |
| Altruistic | 2 | 0 | 8 |
| No-food | 0 | 0 | 8 |
| Pro-social control | 6 | 2 | 9 |
| Altruistic control | 1 | 0 | 9 |

Table S4. Frequency of pulling in the stranger group, averaged across trials.

| **Condition** | **Median** | **min** | **max** |
| --- | --- | --- | --- |
| Selfish | 7 | 0 | 12 |
| Pro-social | 7 | 0 | 11 |
| Altruistic | 0 | 0 | 10 |
| No-food | 0 | 0 | 13 |
| Pro-social control | 8 | 0 | 11 |
| Altruistic control | 0 | 0 | 10 |

# Pre-testing protocol

*Introduction*

- *Room habituation*: The dog was brought to the testing room by two experimenters and was initially allowed a few minutes to explore the room, on both sides of the fence.
- *Noise habituation*: The dispenser produced a loud noise as it dropped the food and it was out of view, therefore the dogs were firstly habituated to the dispenser’s noise. The dog was called at the back of the room by one experimenter, who pressed the remote that operated the food dispensers and immediately dropped a few treats on the floor near the dog. This was repeated two times.
- *Demonstration*: Following the habituation, the experimenters demonstrated the use of the apparatus to the dog. The second experimenter (the trainer) walked up to the apparatus while the first experimenter held the dog. The trainer pulled the trap door open, talking with the dog at the same time to make sure it was watching and could see the food dropping on the floor. The dog was then allowed to go and eat the food. This procedure was repeated throughout the training after each break and during the sessions, to give the dogs a chance so see how the apparatus worked and learn from the trainer’s behaviour.

*First stage (learning)*

- *Toy pulling*: To initially induce the dogs to pull the rope, the trainer played with them using a tug toy, repeatedly feeding the dog a few treats as soon as they tugged the toy. After the first 2-3 repetitions, the trainer stopped any playful behaviour or verbal praise and only rewarded the dog with treats. This was done to ensure that the dog was tugging the toy to obtain the food, rather than play or praise.
- *Rope pulling - helped*: Once the dog was tugging the toy consistently for food, this was attached to the rope on the apparatus, and dogs were given time to practice with it. Initially the trainer held the rope and helped them pulling if necessary, then she gradually intervened less, and moved away from the apparatus.
- *Demonstrations* were also repeated until the dog started pulling the rope. At this point any cueing or prompting to the dog was gradually reduced until stopped.
- *Rope pulling – independent*: Once the dog was able to pull the rope enough that they could open completely the trap door and make the food drop, the trainer gradually walked away from the apparatus and towards the back of the room with the other experimenter.
- *Practice with apparatus*: The dog was then allowed to practice with the apparatus until they could operate it without any cueing, while the experimenters stood at the back of the room, pretending to be distracted. The dos were not required to operate the apparatus as soon as they were released in the room, but if they did not interact with the apparatus within a minute, they were given a break outside of the room.
- *Learning criterion*: this phase was considered concluded when the dog was able to operate the apparatus on its own 5 out of 6 consecutive times, without being given any cue or prompt, while the two experimenters stood at the back of the room pretending to be distracted and ignoring the dog,

*Experimental routine*: The dogs were also regularly led by one of the experimenter at the back of the room and then released, to familiarise them with this part of the testing procedure later on.

*Breaks*: The dogs were give breaks whenever necessary and each training session lasted for up to 1 hour.

*Second stage (knowledge practice)*

- *Practice*: Once the dog reached the learning criterion, the experimenters opened the gate in the fence and started releasing the food from the dispenser placed on the other side of the fence instead than on the dog’s side, so that the dog could experience that food could be delivered on the other side as well. If the dog did not find the food, the trainer would indicate it or direct the dog.
- *Learning criterion*: The dog was let practice with this setting until it reached the same learning threshold as in the learning phase, i.e. the dog was able to operate the apparatus on its own 5 out of 6 consecutive times, without being given any cue or prompt, while the two experimenters stood at the back of the room pretending to be distracted and ignoring the dog.

*Exclusion criteria*

Dogs that did not improve during training for more than 2 consecutive sessions were excluded from the training. Dogs that reached the second threshold were then invited for testing. Dogs were trained on average in 4 training sessions.

# Model diagnostics for the full model, including the interaction between condition and receiver familiarity


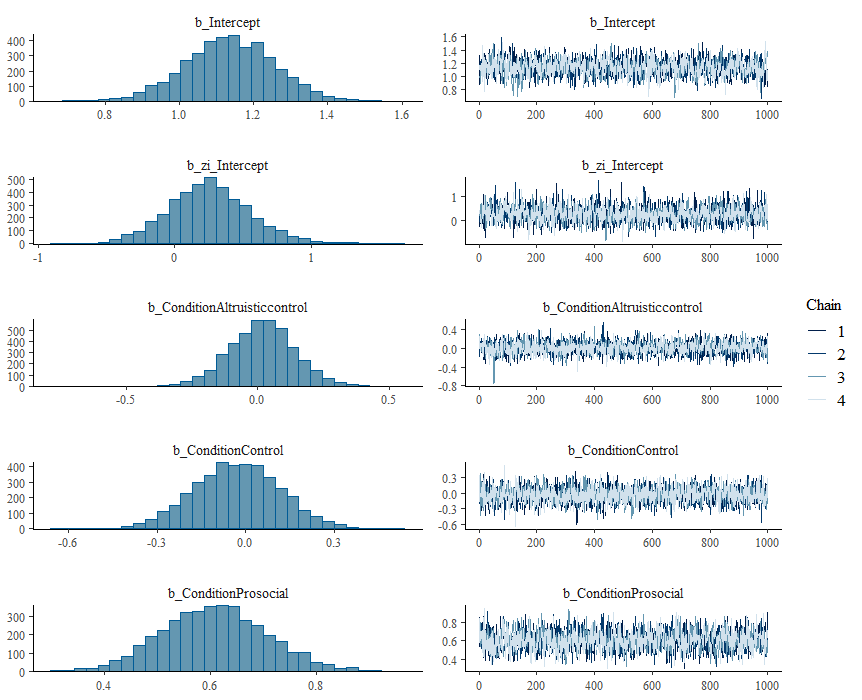


### Figure S1. Trace and density plots of all relevant parameters of the model.


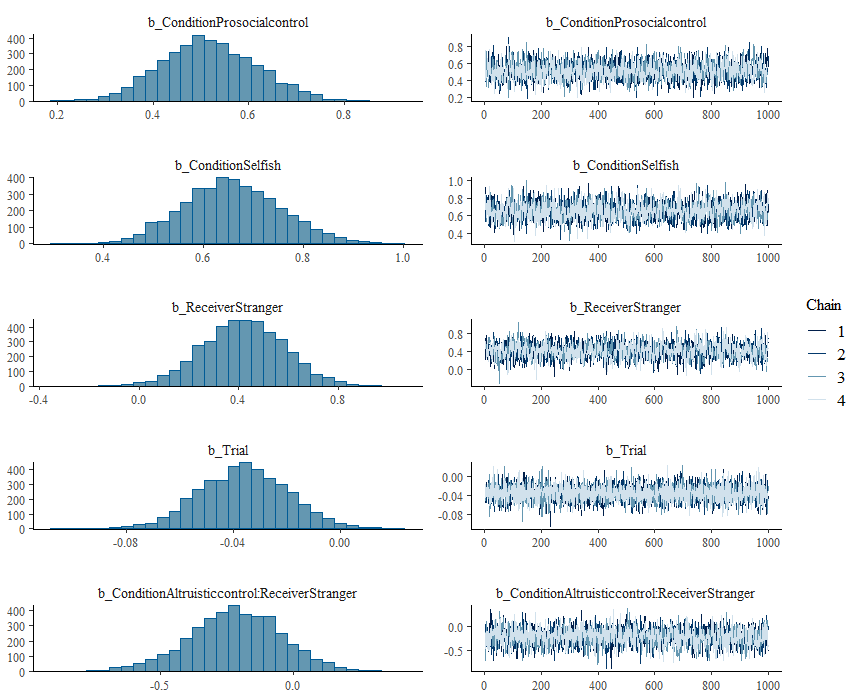


Figure S1 continued. Trace and density plots of all relevant parameters of the model.


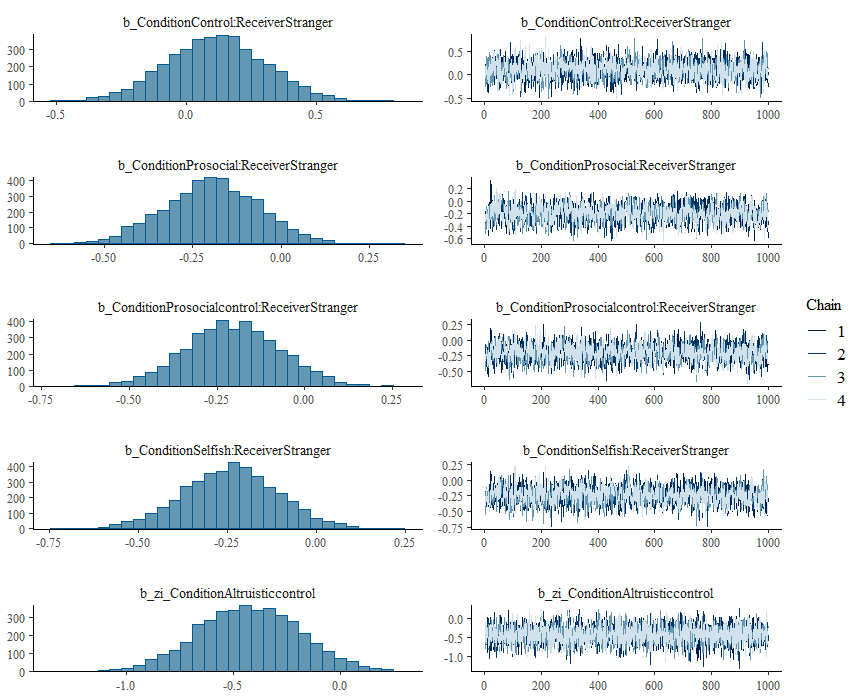


Figure S1 continued. Trace and density plots of all relevant parameters of the model.


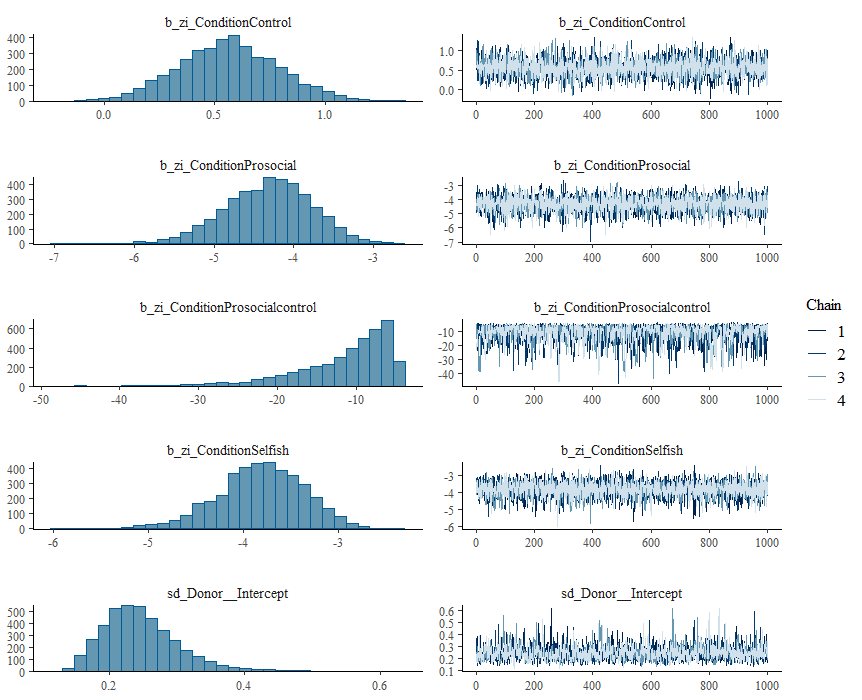


Figure S1 continued. Trace and density plots of all relevant parameters of the model.


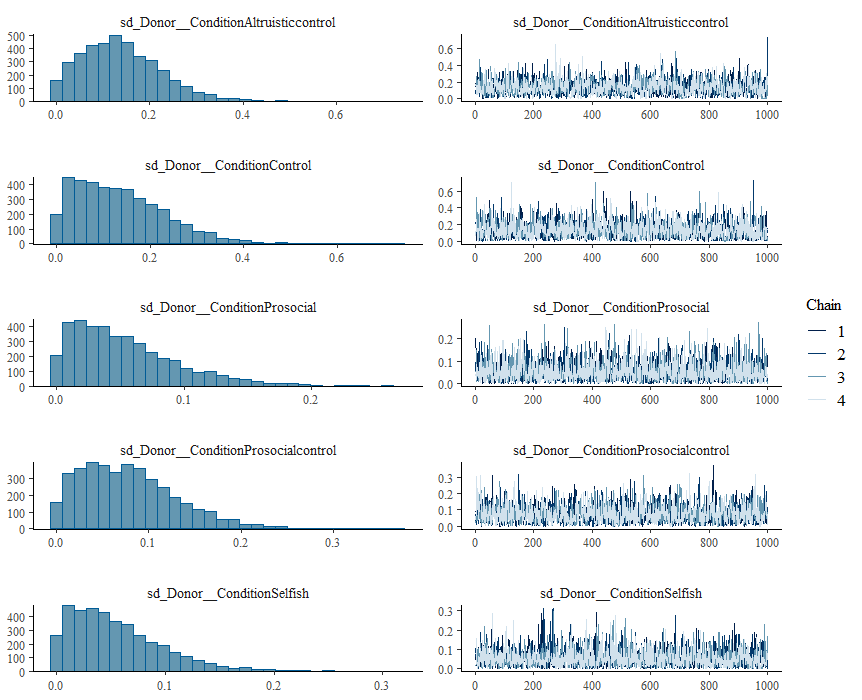


Figure S1 continued. Trace and density plots of all relevant parameters of the model.


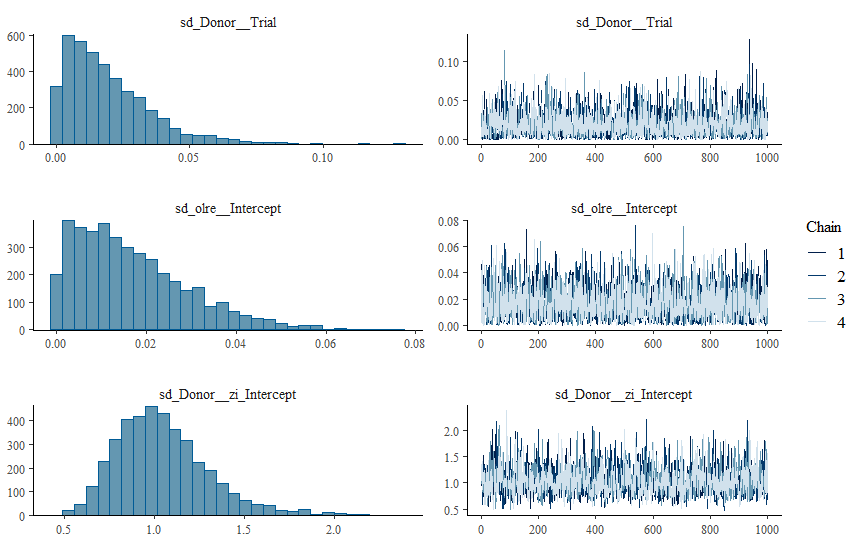


Figure S1 continued. Trace and density plots of all relevant parameters of the model.


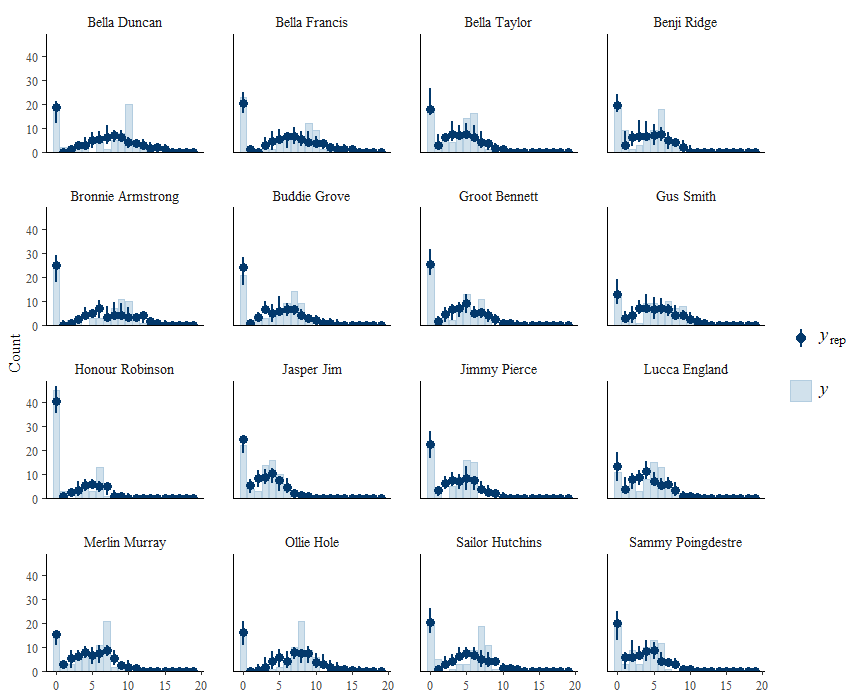


### Figure S2. Plots comparing the observed number of pulls per trial *y* (light blue bars) to 10 simulated datasets *y_rep_* from the posterior predictive distribution (dark blue point and range) for each dog (donor).


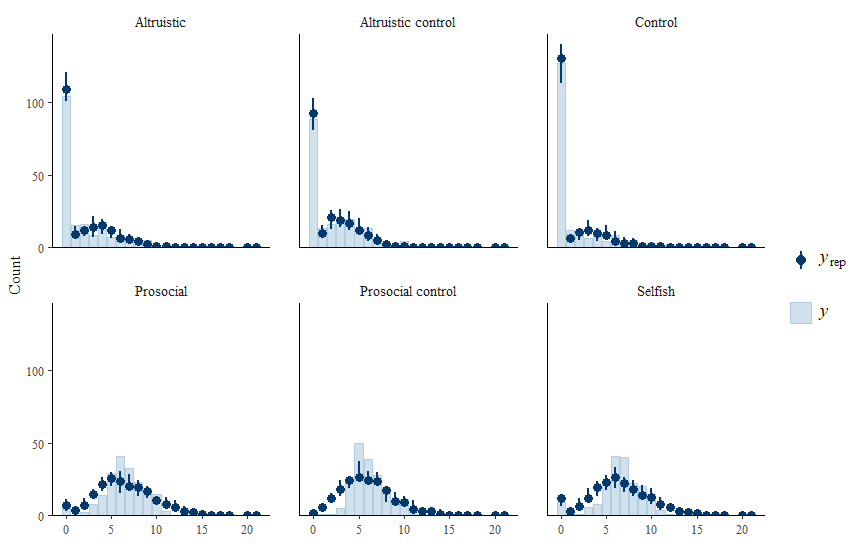


### Figure S3. Plots comparing the observed number of pulls per trial *y* (light blue bars) to 10 simulated datasets *y_rep_* from the posterior predictive distribution (dark blue point and range) in each experimental condition.


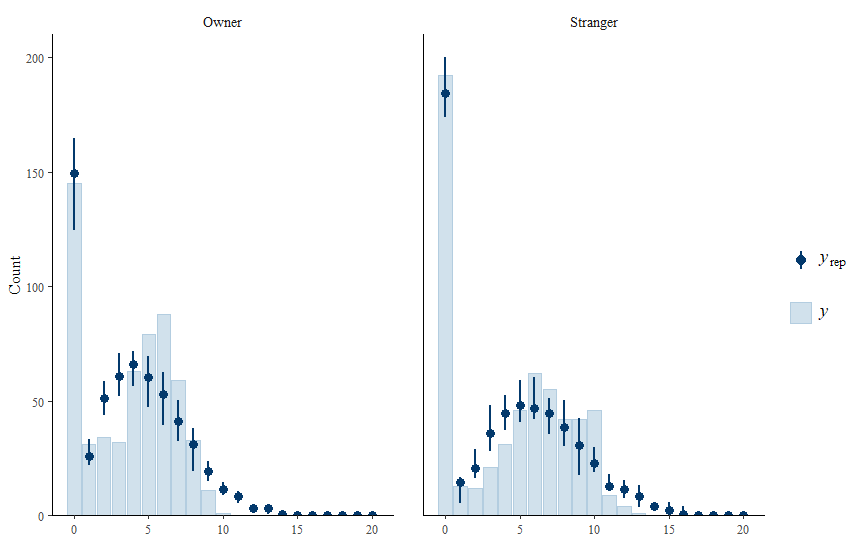


### Figure S4. Plots comparing the observed number of pulls per trial *y* (light blue bars) to 10 simulated datasets *y_rep_* from the posterior predictive distribution (dark blue point and range) for each type of receiver.


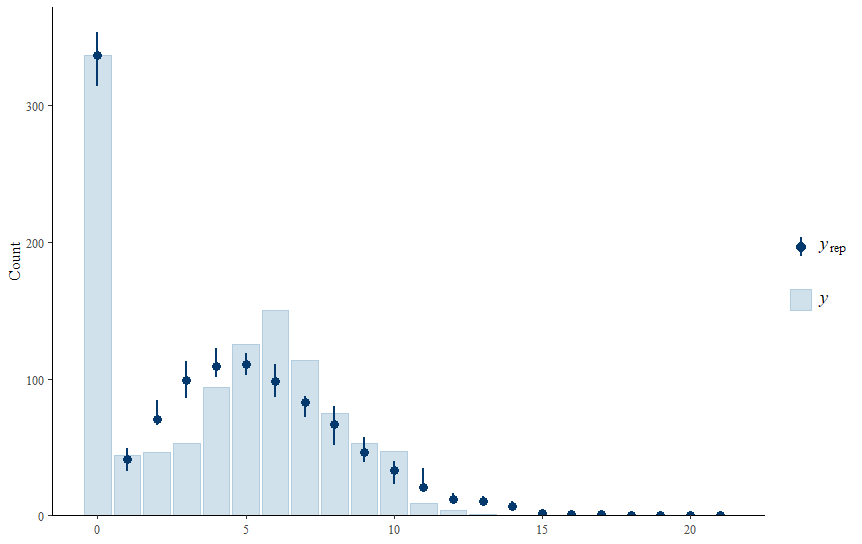


### Figure S5. Plots comparing the observed number of pulls per trial *y* (light blue bars) to 10 simulated datasets *y_rep_* from the posterior predictive distribution (dark blue point and range) for the overall model.

# Model diagnostics for the simpler model, without the interaction between condition and receiver familiarity


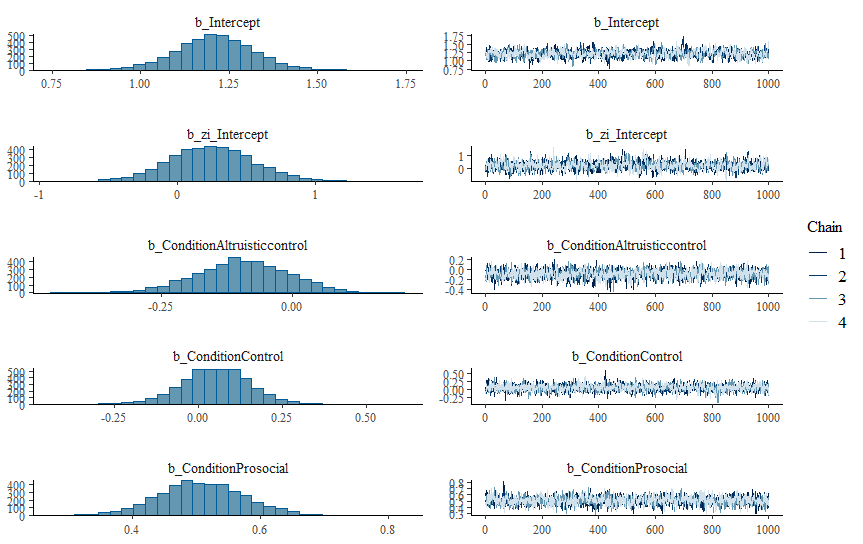


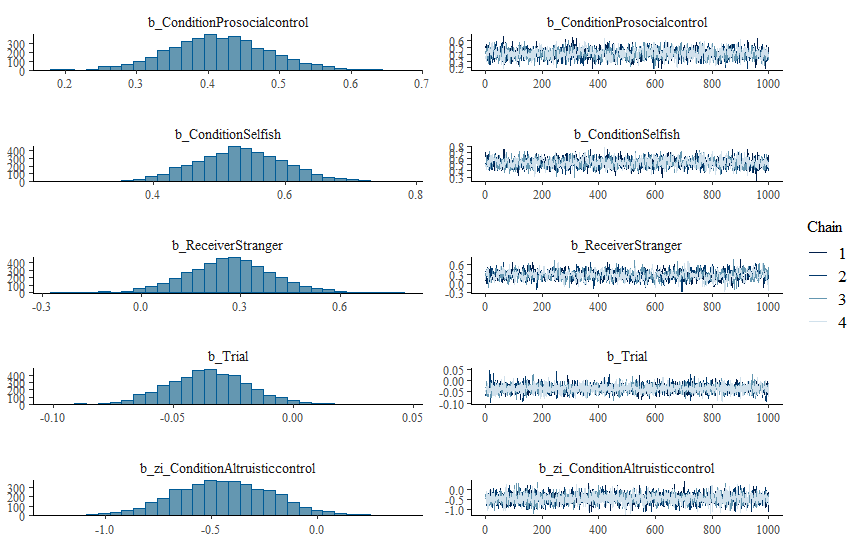


### Figure S6. Trace and density plots of all relevant parameters of the model.


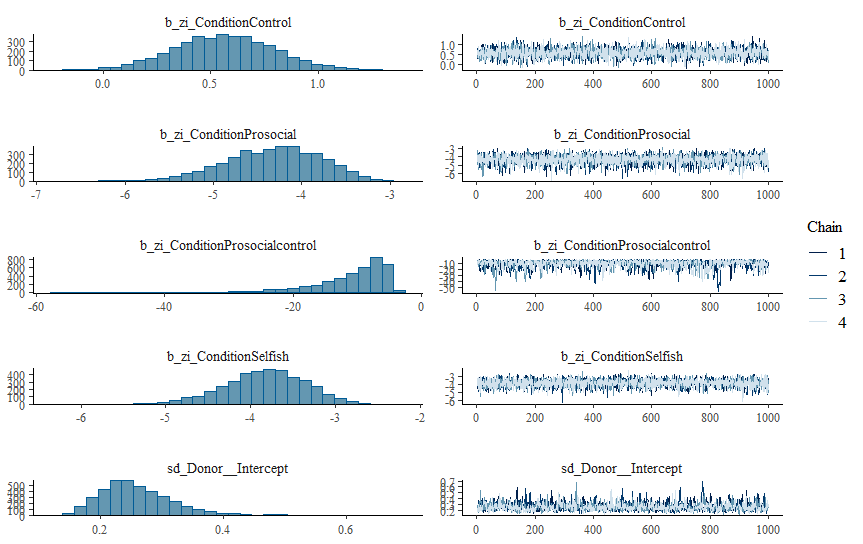


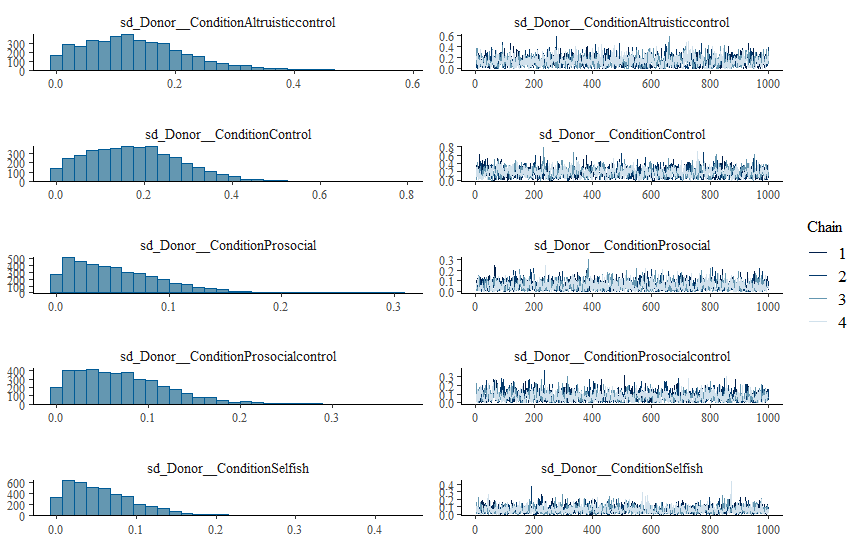


Figure S6 continued. Trace and density plots of all relevant parameters of the model.


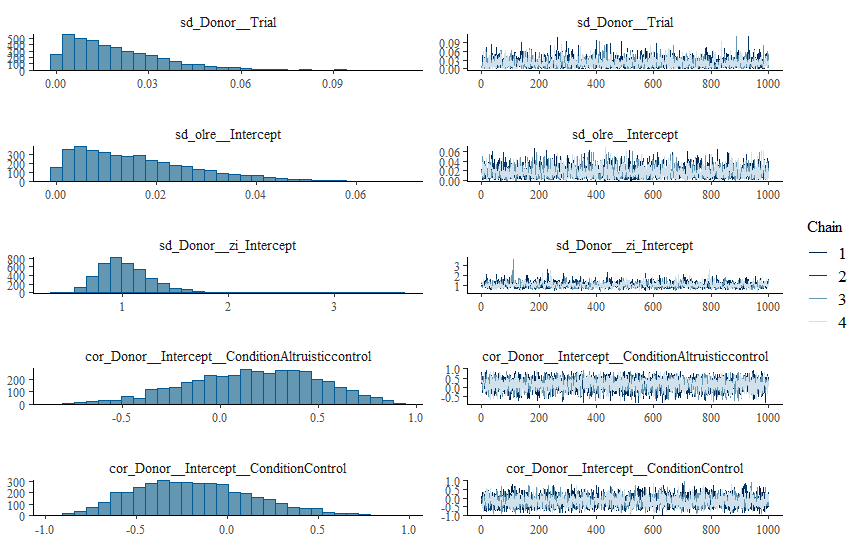


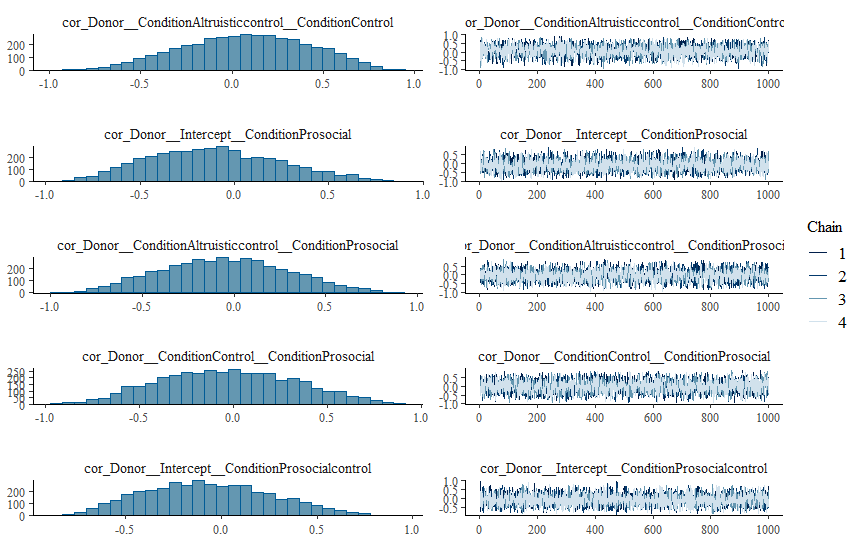


### Figure S6 continued. Trace and density plots of all relevant parameters of the model.


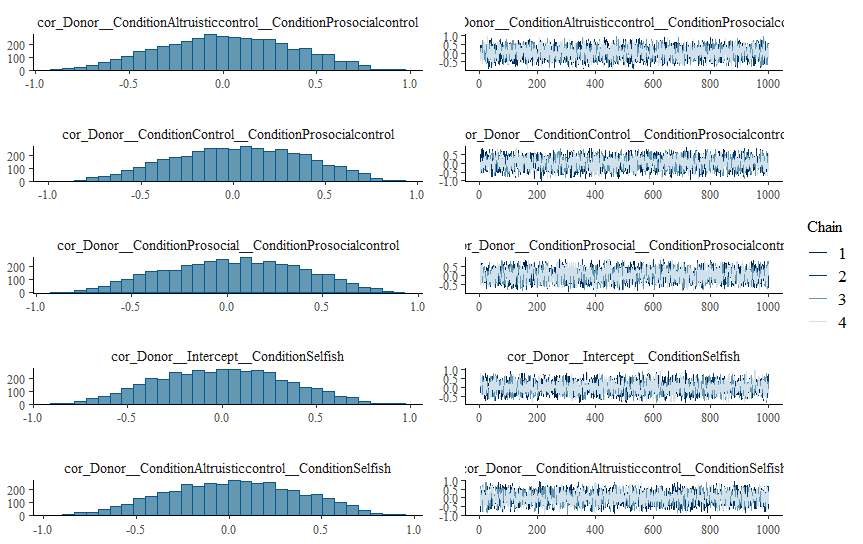


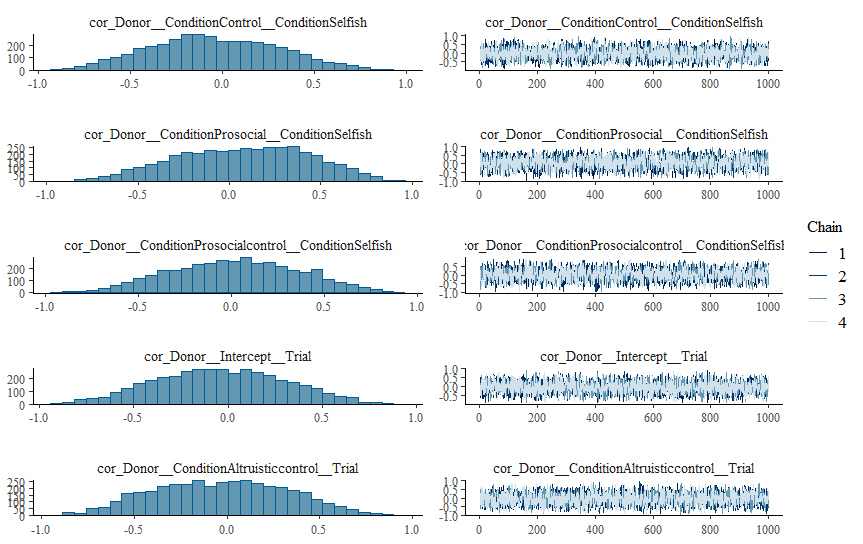


Figure S6 continued. Trace and density plots of all relevant parameters of the model.


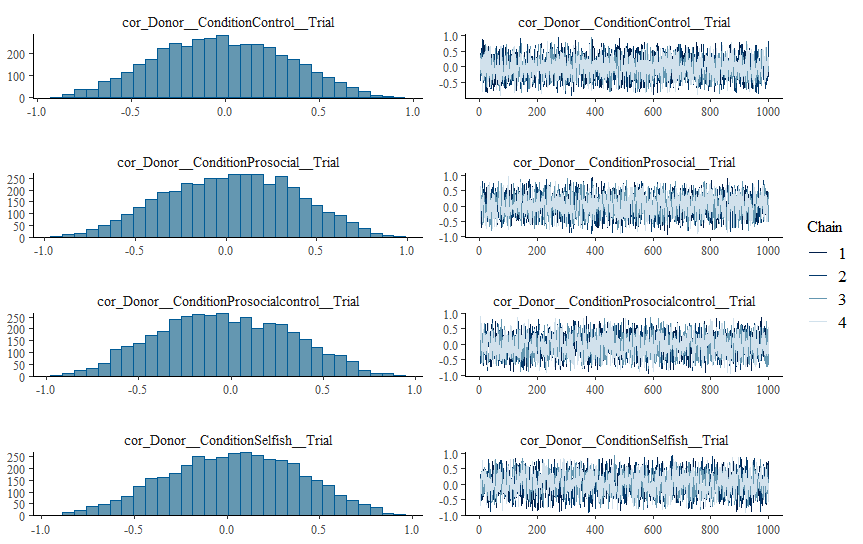


Figure S6 continued. Trace and density plots of all relevant parameters of the model.


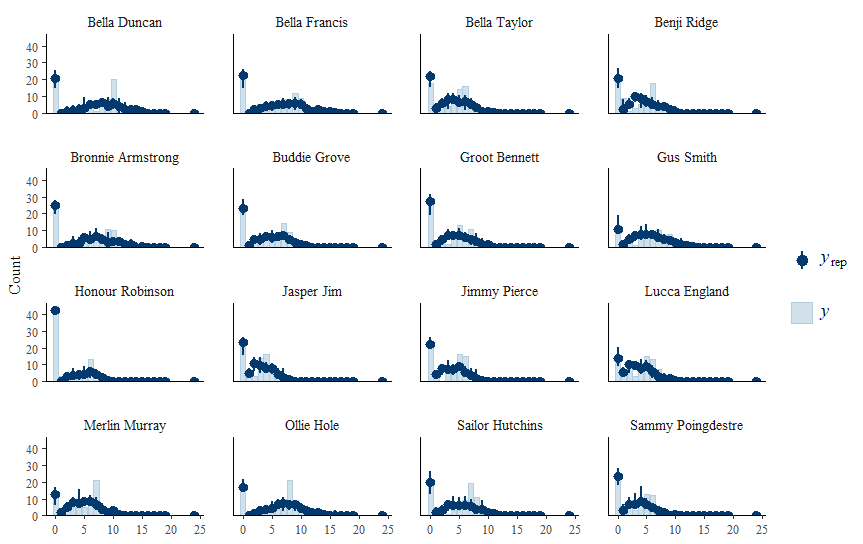


### Figure S7. Plots comparing the observed number of pulls per trial *y* (light blue bars) to 10 simulated datasets *y_rep_* from the posterior predictive distribution (dark blue point and range) for each dog (donor).


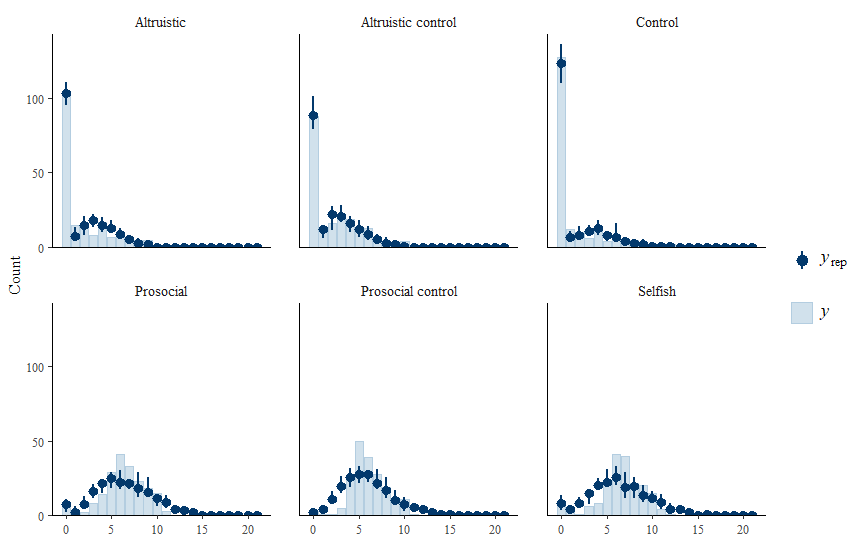


### Figure S8. Plots comparing the observed number of pulls per trial *y* (light blue bars) to 10 simulated datasets *y_rep_* from the posterior predictive distribution (dark blue point and range) in each experimental condition.


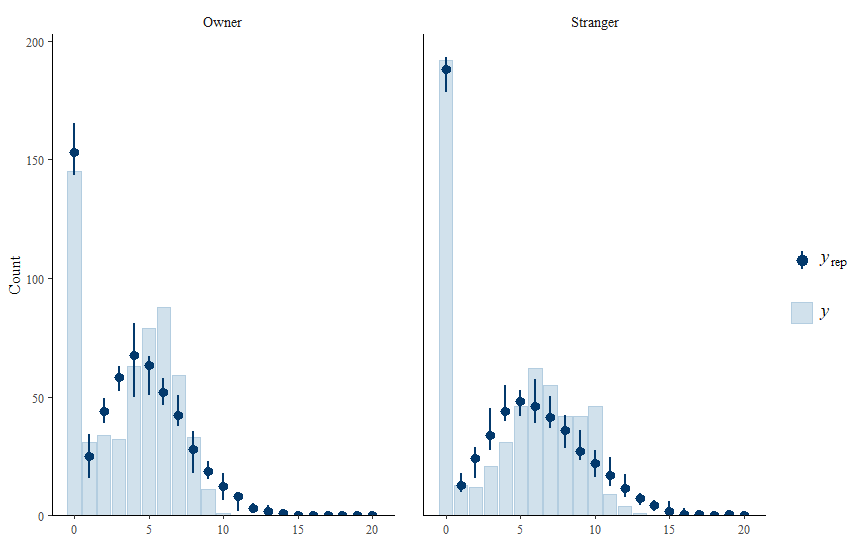


### Figure S9. Plots comparing the observed number of pulls per trial *y* (light blue bars) to 10 simulated datasets *y_rep_* from the posterior predictive distribution (dark blue point and range) for each type of receiver.

###
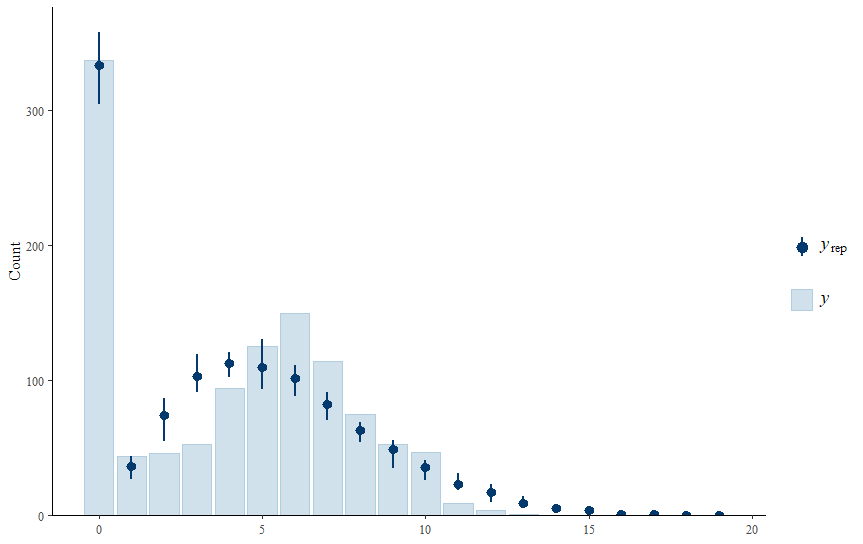


### Figure S10. Plots comparing the observed number of pulls per trial *y* (light blue bars) to 10 simulated datasets *y_rep_* from the posterior predictive distribution (dark blue point and range) for the overall model.

# Model output for full model including an interaction

###

| Effect | Estimate | 95% Credible Interval |
| --- | --- | --- |
| Intercept | 1.14 | 0.90, 1.37 |
| zi Intercept | 0.26 | -0.34, 0.90 |
| Condition |  |  |
| Altruistic | — | — |
| Altruistic control | 0.01 | -0.24, 0.26 |
| Control | -0.02 | -0.34, 0.27 |
| Prosocial | 0.61 | 0.42, 0.80 |
| Prosocial control | 0.52 | 0.33, 0.73 |
| Selfish | 0.66 | 0.47, 0.85 |
| Receiver |  |  |
| Owner | — | — |
| Stranger | 0.43 | 0.10, 0.79 |
| Trial | -0.04 | -0.07, 0.00 |
| Condition:Receiver |  |  |
| Altruistic:Stranger | — | — |
| Altruistic control:Stranger | -0.22 | -0.59, 0.12 |
| Control:Stranger | 0.11 | -0.27, 0.51 |
| Prosocial:Stranger | -0.20 | -0.45, 0.05 |
| Prosocial control:Stranger | -0.21 | -0.48, 0.06 |
| Selfish:Stranger | -0.25 | -0.51, 0.02 |
| zi Condition |  |  |
| Altruistic | — | — |
| Altruistic control | -0.43 | -0.89, 0.01 |
| Control | 0.56 | 0.11, 1.0 |
| Prosocial | -4.3 | -5.4, -3.4 |
| Prosocial control | -12 | -30, -4.7 |
| Selfish | -3.8 | -4.8, -3.0 |

### Table S3. Fixed-effect estimates from the Bayesian zero-inflated Poisson model using brms. The 95% credible intervals are based on posterior distributions.
